# Identifying motivational interviewing techniques in Quitline smoking cessation counselling sessions from Queensland, Australia

**DOI:** 10.1177/13591053241274091

**Published:** 2024-09-01

**Authors:** Hollie Bendotti, Henry M Marshall, Coral Gartner, David Ireland, Sheleigh Lawler

**Affiliations:** 1 Thoracic Research Centre, Faculty of Medicine, The University of Queensland, Australia; 2 Australian e-Health Research Centre, Commonwealth Scientific and Industrial Research Organisation, Australia; 3 Department of Thoracic Medicine, The Prince Charles Hospital, Metro North Hospital and Health Service, Australia; 4 School of Public Health, Faculty of Medicine, The University of Queensland, Australia; 5 NHMRC Centre of Research Excellence on Achieving the Tobacco Endgame, School of Public Health, The University of Queensland, Australia

**Keywords:** behaviour change, counselling, motivational interviewing, smoking cessation

## Abstract

Motivational interviewing (MI) is a common approach for smoking cessation counselling, yet little is known about the use of MI techniques in practice. This qualitative content analysis applied a published classification of content and relational MI techniques to a sample of 30 Quitline transcripts (January-March 2019) from Queensland, Australia. Overall, 36 MI techniques (94.7%) were identified at least once within the total sample. On average, 20 techniques (52.6%) were used in an individual conversation with a small difference observed between initial and follow-up calls. Techniques most frequently applied across conversations were largely relational, while techniques addressing client ambivalence/resistance were less frequently/never applied. Variability in techniques between individual initial and follow-up calls highlights the high degree of personalisation when applying MI to smoking cessation. Further investigations exploring associations of individual techniques and cessation outcomes are warranted. The classification may prove useful for assessments of fidelity for training and monitoring activities.

## Introduction

The onset and maintenance of regular tobacco smoking is influenced by a combination of physiological and behavioural factors (Benowitz, 2010), and therefore smoking cessation intervention often requires a multifaceted approach. Various pharmacotherapies are well established as effective treatments to aid smoking cessation (Hartmann-Boyce et al., 2018; Howes et al., 2020; Lindson et al., 2019a; Mishra et al., 2021) and their effect is increased when used alongside behavioural support (Hartmann-Boyce et al., 2019). Understanding human motivation at the individual-level is central to addressing the complexities of nicotine addiction and supporting successful smoking cessation and relapse prevention (West, 2009). As such, behavioural support is often highly personalised drawing on multiple styles, theories and techniques (Aveyard and Raw, 2012).

Motivational interviewing (MI) is a widely accepted and commonly used psychotherapeutic method for health behaviour change (Frost et al., 2018; Rollnick et al., 2022). MI theory recognises that individuals are the ‘experts of themselves’ and that motivation and behaviour change are elicited internally, with the assistance of MI practitioners to help identify motivations and sustain behaviour change. Interpersonal skills are central and vital to this approach. This is known as the ‘spirit’ of MI and encompasses four main components: collaboration, evocation, autonomy and compassion (Miller and Rollnick, 2012). The MI approach has evolved from two phases (building motivation and consolidating commitment) to four overlapping processes; ‘engaging’, ‘focusing’, ‘evoking’ and ‘planning’ (Miller and Rollnick, 2012), which acknowledge that behaviour change is non-linear, requiring revision or adaptation of plans, commitments and processes as new challenges or barriers often arise. The foundations of MI are reflective listening and four essential communication skills (Open questions, Affirming, Reflection and Summarising). These foundations strengthen the client-centred relationship and act as navigational tools through the focusing, evoking and planning processes (Miller and Rollnick, 2012).

Despite MI being a widely adopted approach to smoking cessation counselling, the evidence regarding its effectiveness is unclear. A 2019 Cochrane Review concluded there is insufficient evidence that MI may or may not assist people in quitting smoking when compared to no intervention or other behavioural interventions, or when used in addition to other behavioural interventions (Lindson et al., 2019b). However, as argued by Davoli and Amato (2014) in the context of harmful alcohol consumption, conventional estimates of population averages for different psychosocial interventions may not provide an accurate assessment of treatment benefit given that patient goals and treatment are highly individualised. Therefore, while more high-quality trials are needed to determine the efficacy of MI for smoking cessation, additional studies to better understand the content and application of MI techniques in smoking cessation counselling are also warranted.

The inherent complexity of MI interventions, while of potential benefit to those seeking support, poses difficulties to researchers attempting to identify specific MI techniques, understand how they are effective in changing behaviour and how they may be replicated. Recognising this issue, Hardcastle et al. (2017) systematically isolated and identified 38 basic MI techniques. Of these, 16 were classified as relational (i.e. interpersonal skills or the MI ‘spirit’) and 22 techniques were classified as content-related (i.e. information/knowledge provided to clients to promote behaviour change). Furthermore, the authors matched 16 MI techniques (14 content and 2 relational) to behaviour change techniques from the Behaviour Change Techniques Taxonomy version 1 (BCTTv1; Michie et al., 2013), suggesting a need for more inclusion of relational techniques within behaviour change taxonomies in general (Hardcastle et al., 2017). While BCTs specific for smoking cessation have previously been identified (Michie et al., 2011), the lack of relational techniques may limit our full understanding of behavioural interventions that incorporate MI. Using this MI classification tool (Hardcastle et al., 2017) to identify content and relational MI techniques specific to smoking cessation counselling may assist in improving the effectiveness and efficiency of these interventions.

Telephone counselling services for smoking cessation, such as Quitline, are an effective option for personalised behavioural support when used alone or in combination with other cessation interventions. Quitline services have been increasingly implemented on a global scale with standardised guides available to train counsellors in the delivery of MI and cognitive behavioural therapy (CBT), and behaviour change techniques (BCTs) alongside the provision of evidence-based education (World Health Organization, 2014). As such, they can also provide valuable insights to better understand the application of tailored counselling techniques for smoking cessation, connected to lived experiences. Quitline calls are frequently audio-recorded, primarily for quality and training purposes. However, this qualitative data repository has been under-used for exploring how health behaviour change interventions are delivered in practice. One study from the United Kingdom found overall low fidelity and high variability in the application of BCTs in telephone behavioural support services, with counsellors thinking they had used more BCTs than were observed (Lorencatto et al., 2014). To our knowledge, there has been no analysis of the application of MI techniques within counselling services for smoking cessation. Filling this knowledge gap by applying a classification tool (Hardcastle et al., 2017) could enhance understanding of how MI techniques are operationalised. In turn, this will inform replicable methods for future research and assessments of intervention fidelity, implementation and effectiveness. Therefore, we aimed to identify and quantify examples of MI techniques within a sample of Quitline counselling sessions completed in Queensland, Australia, by applying a published classification of content and relational MI techniques.

## Methods

### Study design

This study was a qualitative content analysis of Quitline telephone counselling transcripts. We used a published classification tool to identify examples of content and relational MI techniques (Hardcastle et al., 2017). Study method and results are reported following the Standards for Reporting Qualitative Research (SRQR; O’Brien et al., 2014).

### Ethical considerations

Ethics approval was granted by The Prince Charles Hospital Human Research Ethics Committee (Project ID: 50620). Access to Quitline audio files was obtained following agreement by the Quitline data custodian (Queensland Health), and an approved waiver of consent via a *Public Health Act 2005* (PHA 50620) application.

### Sampling and data collection

Quitline (Queensland Health) selected and provided a purposive sample of 30 audio files for 30 individuals based on demographic characteristics including age, sex, rural/remote location and Indigenous status (Table 1). This approach was requested by the study team to ensure diversity in our sample and inclusion of priority populations for smoking cessation. Audio files were manually transcribed verbatim and de-identified prior to analysis. Within the total sample, 26 were initial calls and four were support (follow-up) calls, completed by 23 individual counsellors (Female: *n* = 21) between January and March 2019. Qualification levels and experience of Quitline counsellors was not recorded, but in Queensland, the majority (>87%) have tertiary qualifications in psychology or social work and those without either have a diploma qualification or are actively undertaking relevant tertiary education. Nevertheless, all Quitline counsellors, regardless of prior qualifications, complete standardised smoking cessation training, onboarding, monitoring and professional development activities. In Australia, basic training for Quitline counsellors is consistent with the World Health Organization (WHO) manual *Training for tobacco quitline counsellors: telephone counselling* which includes MI, CBT and BCTs (Cancer Council Victoria, 2024; World Health Organization, 2014). Type of call was not known during purposive sampling and was identified during analysis, and support calls were not linked to included initial calls. The length of conversations ranged from 16 to 56 minutes (*M* = 36 minutes). The mean age of the Quitline clients was 44.3 years (*n* = 29), about half were female (*n* = 16) and most lived in a regional/rural area (*n* = 19).

**Table 1.** Summary of client demographics and current smoking behaviour.

Characteristic	Value
Age (years), mean (SD)^a^	44.3 (13.4)
Sex, *n* (%)	
Male	14 (47)
Female	16 (53)
Location	
Metropolitan	11 (37)
Regional/rural	19 (63)
Aboriginal and/or Torres Strait Islander, *n* (%)^b^	3 (10)
Smoking status	
Currently smoke	26 (87)
Quit	4 (13)
Cigarettes per day (pre-quit), mean (SD)^c^	16.5 (6.9)
Age started smoking (years), mean (SD)^d^	16.2 (3.5)

a Missing data *n* = 1.

b Missing data *n* = 18.

c Missing data *n* = 3.

d Missing data *n* = 7.

### Analysis

Transcripts were imported into NVivo (Version 12) for qualitative content analysis. Deductive coding followed a published classification of content and relational MI techniques (Hardcastle et al., 2017) that summarises techniques by process stage and identifies MI techniques that are equivalent to BCTs in BCTTv1 (Michie et al., 2013). Analysis was completed by two study team members (senior researcher and early career researcher) with formal training and experience in qualitative research as well as expertise in health psychology, health promotion and public health. Study team members (HB & SL) coded a random sample of transcripts (*n* = 5) together to ensure and establish agreement of interpretation of MI technique definitions (Hardcastle et al., 2017) via in-depth discussion. The remaining transcripts (*n* = 25) were then coded by manual examination and classification by one team member (HB), with SL available to discuss examples when required. Collated examples were reviewed by SL and discussed and agreed upon by consensus between both researchers. Following combined review, four examples were excluded across three techniques. The frequency of MI techniques was quantified, and descriptive statistics calculated, for individual transcripts and across the total sample. Differences in MI techniques between initial and support calls were examined as they commonly reflect varying stages of behaviour change for smoking cessation and thus serve different purposes.

## Results

Identified MI techniques and their frequencies across Quitline conversations are presented in Table 2. Overall, 36 out of 38 MI techniques (94.7%) were identified at least once within the total Quitline sample. This was consistent among initial calls, and 31 MI techniques (81.6%) were identified at least once within follow-up calls. MI techniques unique to initial calls included: *5. Agenda mapping*, *7. Permission to provide information and advice*, *15. Hypothetical thinking* and *23. Double-sided reflection.* The techniques frequently applied within all conversations (i.e. coded 100%) were mostly relational and included *1. Open-ended questions*, *2. Affirmation*, *3. Reflective statements*, *20. Troubleshooting* and *35. Support change/persistence.* Several techniques were never or infrequently applied. These techniques tended to address client ambivalence or resistance, including *25. Overshooting* (*n* = 0), *28. Shifting focus* (*n* = 3, 10%) and *27. Coming alongside* (*n* = 3, 10%), but also *34. Goal attainment scaling* (*n* = 0), *15. Hypothetical thinking* (*n* = 3, 10%), *14. Looking back* (*n* = 4, 13%) and *13. Looking forward* (*n* = 4, 13%).

**Table 2.** Frequency of MI techniques identified in Quitline sample and within individual transcripts (Hardcastle et al., 2017).

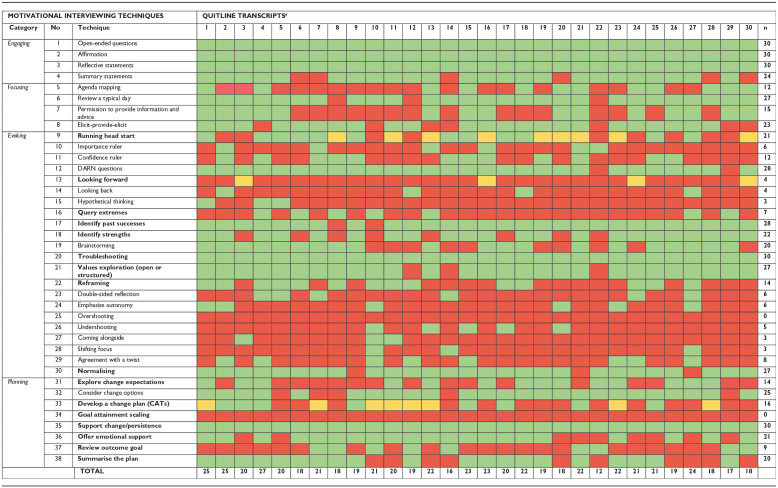

Techniques in bold overlap with behaviour change techniques from BCTTv1. Identified (Green); Partially completed (Yellow); Not identified (Red).

a Support calls: Transcripts 8, 10, 12, 22.

Mean frequencies of MI techniques are presented in Table 3. Across the sample, an average of 20 MI techniques were identified per conversation. Initial calls used a slightly higher mean number of techniques (mean 21 (SD 2.69; Min 16, Max 27)) compared to support calls (mean 18 (mean 3.87; Min 12, Max 21)). When considering MI techniques by process stage across all calls, on average, less than half of *Evoking* techniques were identified, while *Engaging* techniques were frequently used. *Focusing* techniques were less frequently used in support calls (mean 1 (SD 0.5; Min 0, Max 1)) compared to initial calls (mean 3 (SD 0.78; Min 1, Max 4)), and average use of *Planning* techniques was similar between the types of calls. Additionally, the mean frequency of content and relational MI techniques was similar overall and between initial and support calls.

**Table 3.** Mean frequencies of MI techniques.

Item	Total calls (*N* = 30)	Initial calls (*n* = 26)	Support calls (*n* = 4)
MI techniques^a^ per session, mean (SD; min, max)	20 (3.01; 12, 27)	21 (2.69; 16, 27)	18 (3.87; 12, 21)
MI techniques by MI process stage, mean (SD; min, max)			
*Engaging* (*n* = 4)	4 (0.41; 3, 4)	4 (0.42; 3, 4)	4 (0; 4, 4)
*Focusing* (*n* = 4)	3 (1.04; 0, 4)	3 (0.78; 1, 4)	1 (0.5; 0, 1)
*Evoking* (*n* = 22)	9 (2.08; 4, 14)	10 (1.89; 7, 14)	8 (2.94; 4, 11)
*Planning* (*n* = 8)	5 (1.04; 2, 6)	4 (1.07; 2, 6)	5 (0.96; 4, 6)
BCT/MI techniques^b^ per session, mean (SD; min, max)	10 (1.40; 6, 13)	10 (1.29; 7, 13)	9 (1.89; 6, 10)
Relational techniques^c^ per session, mean (SD; min, max)	8 (1.41; 5, 11)	8 (1.11; 6, 10)	7 (2.71; 5, 11)
Content techniques^d^ per session, mean (SD; min, max)	12 (2.28; 7, 17)	13 (2.16; 8, 17)	11 (2.65; 7, 13)

Mean values rounded to whole numbers.

a *N* = 38.

b *N* = 16.

c *N* = 15.

d *N* = 23.

Qualitative examples of BCT/MI techniques (and their definitions) are outlined in Table 4. Of these techniques, *34. Goal attainment scaling* was never identified within the total sample. The BCT/MI techniques of *9. Running head start*, *13. Looking forward* and *33. Develop a change plan* were sometimes only partially completed based on the definition. On average, 10 BCT/MI techniques were used per Quitline conversation, with little variation between initial and follow-up calls (Table 3).

**Table 4.** Qualitative examples of motivational interviewing techniques that overlap with behaviour change techniques (BCTTv1).

Technique	Definition (Hardcastle et al, 2017)	Quitline example
Running head start (Con)	A strategy for eliciting client motivational talk in which the counsellor asks open questions to first explore the perceived ‘good things’ about the status quo, in order to then query the ‘not so good things’ about the status quo.	P: ‘*When you smoke you feel like, it kind of helps manage the depression? …* C: *So, you notice it’s taking more of a toll on your breathing and your health now that you’re older?*’ [T1]
Looking forward (Con)	The client is prompted to envision two possible futures. The first ‘future’ is if they continue on the same path without any changes where they might be 5 or 10 years from now. The second future is if they decide to make a change, what their future might look like.	C: ‘*What are you looking forward to as someone who doesn’t smoke*?’ [T3] (Partially completed)
Query extremes (Con)	A technique used to evoke change talk by asking clients to imagine best consequences of change or worst consequences of status quo.	C: ‘*Have you noticed any benefits yet to being smoke free for the last few days?*’ [T8]
Identify past successes (Con)	The counsellor prompts the client to think about previous successes at behavioural changes to build confidence for change.	C: ‘*… what did you do when you would quit for those three months? How do you change?*’ [T16]
Identify strengths (Con)	The counsellor prompts the client to draw out their strengths and the relevance of these strengths to making successful behavioural changes.	C: ‘*I think the really good thing REDACTED is that you’re actually really insightful about what the triggers are for you to actually feel like a smoke. And the best thing about that is when you’re a conscious smoker like you are, it’s great ‘cause you can build strategies around what you’ll do different*’ [T5]
Troubleshooting (Con)	The counsellor prompts the client to think about potential barriers and identify ways of overcoming them in order to strengthen motivation.	C: ‘*Are there any particular challenges that you’re envisioning?*’*…*‘*What sort of coping mechanism could you use?*’ [T30]
Values exploration (open or structured; Con)	The counsellor prompts the client to explore his or her values and how the behaviour fits in with these values. The counsellor may ask the client to describe their main goals and values in life.	C: ‘*You’ve been trying a few times, what’s behind that? Why do you want to keep quitting? What’s motivating you?*’ [T23]
Reframing (Con)	A counsellor reflective statement that invites the client to consider a more positive and motivational interpretation of what has been said.	P: ‘*What I don’t wanna* (sic) *be is in a week in a half times, back to smoking 40 cigarettes in a day … And the problem I’ve got at the moment is the feels that it’s building up and building up*’. C: ‘It’s that taking the little bit of control back’. [T11]
Normalising (Con)	The counsellor communicates to clients that having difficulties while changing is not uncommon	C: ‘*You said you noticed you felt like you’re umm, I think the word you used was felt like you were going a bit crazy … So that’s very normal, very common … It’s basically withdrawal symptoms*’. [T25]
Explore change explorations (Con)	The counsellor prompts the client to identify the outcomes that the client expects to achieve based on the changes that they are motivated to make.	C: ‘*… what are you hoping would come from quitting?*’ [T20]
Develop a change plan (CATs; Con)	The counsellor prompts the client to develop a specific change plan that the client is motivationally ready to accept.	C: ‘*Do you have any goals that you would like to set for yourself for when we call you in 3 weeks time?*’ [T12]
Support change/persistence (Rel)	The counsellor functions as a partner or companion, collaborating with the client’s own expertise.	P: ‘*And my motto was, I’m not gonna* (sic) *stop giving up, give up*’. C: ‘*That’s the perfect motto for quitting REDACTED*’. [T14]
Offer emotional support (Rel)	The counsellor offers reassurance, to the client.	C: ‘*You’re doing fantastic to keep it as low as you have considering all that you’re going through so that’s gonna* (sic) *give you confidence in going into the future*’. [T11]
Review outcome goal (Con)	The counsellor asks the client how they are progressing with their goals.	C: ‘*How’s your quit journey been going?*’ [T22]
Summarise the plan (Con)	The counsellor summarises the change plan including the specific behavioural goals, the reasons for making the change, the specific steps to be taken, the outcome goals and coping planning for relapse prevention.	C: ‘*We’ll let you source your patches and your lozenges …. Give some thought to some of those strategies. I’ll pop a little booklet in the mail to you that has some nice ideas in it … Then we can schedule you a call whenever you want just to check in and see how you’re travelling*’. [T24]

(Con): content; (Rel): relational; [T]: transcript number; C: counsellor; P: patient.

## Discussion

This is the first content analysis of MI techniques used in telephone smoking cessation counselling conversations. Results indicate that most techniques were applied across the entire Quitline sample, yet at the individual conversation level, only half of the range of techniques, on average, were applied. This highlights the variability in the content of smoking cessation counselling sessions. Individual needs, knowledge, preferences and level of engagement and motivation guide the conversation, meaning counsellors are reflective in their style or approach to suit the client, and may not need to use the full range of techniques available to them for every conversation. Such an approach is consistent with the WHO Training for Tobacco Quitline Counsellors in that basic MI skills (Open questions, Affirming, Reflection and Summarising) are employed alongside strategies for applying the principles of MI (express empathy, develop discrepancy, roll with resistance, support self-efficacy), and models for conducting MI such as the 5Rs model (Relevance, Risks, Rewards, Roadblocks, Repetition; World Health Organization, 2014).

Initial and follow-up calls serve different purposes and yet we found little difference in the total number of MI techniques used between them. The types of techniques unique to initial calls related to educating, assessing behaviour/s prior to change, eliciting ideas about behaviour change and capturing clients’ reasons for and against change. Similarly, *Focusing* techniques were more frequently observed in initial calls compared to follow-up calls as these techniques explore the direction and goal/s of the client. This is consistent with the evocation of ‘change talk’ that is a unique feature of MI (Miller and Rollnick, 2012). Understandably, evoking ‘change talk’ is more pronounced in initial calls as counsellors seek to elicit and encourage motivation to set clients up on their quit journey, whereas follow-up calls are an opportunity to sustain ‘change talk’ by reviewing the plan and goals already in place.

There was little use of *Evoking* techniques that address client negativity, resistance and/or low levels of motivation. This may be indicative of where the average Quitline client lies within the Stages of Change (SOC) Model (Prochaska and Velicer, 1997) in terms of readiness to change their smoking behaviour, but also that individual negativity and low motivation may have precluded any engagement with the service whatsoever, that is, individuals with low quit motivation declined the offer of Quitline counselling or were never referred. Given that Quitline is a service people can directly reach out or accept a referral to, there is an assumption that individuals may be at the contemplation, preparation or action stages of change upon initial contact with Quitline and thus would have a level of motivation. Furthermore, combining the SOC with MI is recognised as a potentially useful strategy to better tailor the MI intervention based on stages of motivational readiness (Miller and Rollnick, 2004; Petrocelli, 2002). Client negativity that does arise within counselling sessions may be due to low expectations related to previous unsuccessful attempts, which is a common experience when quitting smoking (Chaiton et al., 2016; Steinberg et al., 2008). The minimal use of these certain techniques may also be a reflection of the Quitline counsellors’ ability to reduce ‘sustain talk’ or arguments for maintaining the status quo and resisting change (Miller and Rollnick, 2012).

Relational techniques during the engaging and planning processes were consistently applied across most conversations. These are important to build rapport and a positive working relationship, and to create an environment that allows the client to feel safe and comfortable. Previous research has identified client ambivalence to engaging with telephone-based services due to cognitive barriers such as stigma, variable expectations and discomfort (Albert et al., 2020; Solomon et al., 2009). Therefore, there is a need for a strong relational approach to engagement to elucidate and address any pre-conceived ideas of first-time clients or returning clients hesitant to re-engage. As mentioned previously, there was little crossover of relational MI techniques with the BCTTv1 as it is largely content focused (Hardcastle et al., 2017). If we had only applied a general behaviour taxonomy to our analysis as a classification guide, we would have omitted the essential ‘spirit’ of MI which guides and allows for discussion of the practical elements of quit plans.

The MI technique definitions developed by Hardcastle et al. (2017) served as a useful tool to isolate techniques used within our sample. Other studies have found this tool to be reliable and feasible in evaluating the application of MI techniques in diabetes prevention (MacPherson et al., 2020) and physical activity counselling (PAC; Gagnon et al., 2018). Our study extends this work being the first to apply it to smoking cessation counselling. Our analysis identified more MI techniques across the sample and a higher average number of MI techniques were used per session compared to previous studies examining MI use for diabetes prevention (MacPherson et al., 2020) and PAC (Gagnon et al., 2018), but *Engaging* techniques predominated all analyses highlighting consistency in a strong relational approach to health behaviour counselling. A classification tool of this nature allows for replication and comparison of evaluation studies of behaviour change interventions, as well as better identification of techniques in relation to impact on health outcomes. Future research could seek to formally validate this tool as a reliable method for evaluating the fidelity of MI interventions, including Quitline training or monitoring processes.

A key strength of our study is that we have analysed a diverse sample of Quitline counselling conversations that were not elicited for research purposes. This limits the influence of the Hawthorne effect (Allen and Davis, 2011), however we cannot ascertain whether information provided by clients to counsellors was entirely accurate. Such a dataset provides practical knowledge and examples of the smoking cessation counselling process, and insight into the lived experiences connected to health behaviour change processes. The purposive sampling of Quitline calls may introduce selection bias, however selection of call was based on demographic groups, which were largely balanced, and not by individual dialogue content. Limitations to our study also include the potential that variation in the application of MI may have been influenced by differences in training and experience between counsellors. Initial and support calls were unbalanced within the sample which limits our ability to definitively compare MI techniques between type of calls. We also lack outcome data and program completion rates associated with the included Quitline clients. Linking outcome data to the application of MI techniques in Quitline counselling was beyond the scope of this study and would require an analysis of the entire client journey (i.e. subsequent conversations) that also accounts for potential confounders (e.g. pharmacotherapy use). While our findings should be interpreted with caution as we are unable to determine the potential effect of specific MI techniques or processes, this exploratory study sets a precedent for future research in this area using this published MI classification tool as the reference. The practical application of this published MI classification tool (Hardcastle et al., 2017) to reliably report the active content and relational intervention components in smoking cessation counselling with engaged clients improves our understanding of how these MI techniques are operationalised which can inform counselling intervention developments, and future research and assessments of intervention fidelity and implementation.

## Conclusion

In this sample of Quitline counselling sessions, relational and content MI techniques were frequently used but varied in frequency and types reflecting a high degree of tailoring to the individual. MI techniques were successfully identified using a published classification tool, which allows for consistent evaluation, replication, translation and comparison with other health behaviour change interventions. Further research should explore associations between the use of MI techniques relative to patient characteristics and smoking cessation outcomes, to better understand the effect of individual components of behavioural interventions.
